# Factors Related to Relapse of Congenital Talipes Equinovarus (CTEV) After the Ponseti Method

**DOI:** 10.7759/cureus.43701

**Published:** 2023-08-18

**Authors:** Waleed A Mohsenh, Mahdi M Alqarni, Abdullah K Alshehri, Abdullah M Asiri, Ohood H Mohsenh, Syed E Mahmood, Ali I Alhifzi, Reem H Mohsenh, Ahmed S AL Zomia

**Affiliations:** 1 Department of Orthopedic Surgery, Aseer Central Hospital, Abha, SAU; 2 Department of Pediatric Orthopedic, Abha Maternity and Children Hospital, Abha, SAU; 3 College of Medicine, King Khalid University, Abha, SAU; 4 Department of Family and Community Medicine, King Khalid University, Abha, SAU; 5 Department of Orthopedic Surgery, Aseer Central Hospital, King Khalid University, Abha, SAU; 6 Intensive Care Unit, Aseer Central Hospital, Abha, SAU

**Keywords:** factors, risk, ponseti, relapse, ctev

## Abstract

Background: Congenital talipes equinovarus (CTEV) is one of the common congenital disorders in pediatric orthopedic practice that affects a large group of children.It is a combination of four parts of deformity that affect either a single foot or both feet. Our aim in this study is to estimate the prevalence and incidence of CTEV and to evaluate the risk factors that lead to relapse in some children to avoid relapse in future and complex surgical interventions, as well as to improve the final outcome.

Materials and methods: A retrospective cohort study for the cases of CTEV was conducted to estimate the prevalence of relapse in children with CTEV after management by the Ponseti method and to evaluate the risk factors that lead to recurrence.

Result: The study includes 103 patients with CTEV, and only 22 patients had relapse. The prevalence rate of relapsed cases was 20.4%, and the incidence was 42 per thousand. The average number of casts applied was 4.05 ± 1.37. The average severity of the deformity that was measured by the Pirani score was 4.97 ± 1.21. The most common atypical presentation of CTEV was associated with developmental dysplasia of the hip (DDH), followed by myelomeningocele (MMC).

Conclusion: The only significant factors in the study were the Pirani score and non-compliance of the brace with p < 0.05. There was not any significance in the correction of the deformity by Ponseti between idiopathic and non-idiopathic CTEV based on the number of casts and the Pirani score. The dynamic foot brace can be the solution for the high recurrence rate, yet more studies are needed in the future.

## Introduction

Congenital disorders, in general, are difficult to estimate and predict in one single population, due to their variety and complexity. Furthermore, some congenital disorders affect more than one organ or system. However, congenital talipes equinovarus (CTEV) is one of the common congenital disorders in pediatric orthopedic practice that affects a large group of children [1]. It is a combination of four parts of deformity that affect either a single foot or both feet. First, cavus deformity happened due to tightness of intrinsic muscles of the foot with tight flexor hallucis longus and flexor digitorum longus muscles. Second, another muscle, which is the posterior tibialis muscle, will make the foot in an adduction position and varus along with tibialis anterior and tendoachilles. Finally, equinus will happen at the end due to tendoachilles tightness [2]. There are two types of CTEV: idiopathic, which is more common, and non-idiopathic, which is usually present with other congenital disorders. Atypical presentation of CTEV means that patients will present with not only CTEV but also with other congenital disorders, such as developmental dysplasia of the hip (DDH), myelomeningocele (MMC), metatarsus adductus, and other syndromes. Our study shows that almost 25% of patients with atypical neurogenic atypical presentation will have relapse, compared to 20% of patients with non-neurogenic atypical presentation, which does not show any significant finding in atypical presentation in terms of relapse [3,4]. Multiple studies worldwide have agreed on a high number of recurrence rates of CTEV after management by the Ponseti method, which ranges from 10% to 40% [2-4]. The need for evaluation of the risk factors that lead to the recurrence becomes mandatory. However, the most significant risk factor, which was mentioned in all articles, is the non-compliance of a brace [5-7]. In our referral clinic of pediatric orthopedics, we manage frequent cases of CTEV; hence, we are interested to study the risk factors of relapse. Nevertheless, the rate of relapse after treatment of the Ponseti method has not been studied yet in our region. Our aim in this study is to estimate the prevalence and incidence of CTEV and to evaluate the risk factors that lead to relapse in some children to avoid relapse in the future and complex surgical interventions, as well as to improve the final outcome.

## Materials and methods

A retrospective cohort study for the cases of CTEV was conducted to estimate the prevalence of relapse in children with CTEV after management by the Ponseti method and to evaluate the risk factors that lead to recurrence. Ethical approval was obtained from the regional committee for research ethics. Consent was not needed from patients. The list of patients was provided by the Department of Pediatric Orthopedic after obtaining the institutional review board approval. The research team took the responsibility of collecting the data from the patients' file. All patients underwent a weekly serial casting in our Ponseti-assigned clinic, and percutaneous tendoachilles tenotomy was done on most of the patients. The number of sessions depends on the severity and progress of improvement. Then, a foot abduction brace (Denis Brown shoes) was used. A foot abduction brace should be applied for 23 hours in the first 12 weeks after the serial casting, and then it can be decreased to 18 h in the second 12 weeks. The severity of the deformity was assessed by the Pirni score and documented in the files. The reappearance of one of the four components of CTEV was defined as recurrence.

Study setting

The study took place at a governmental hospital, with a capacity of 240 beds, delivering comprehensive care to both Saudi citizens and foreign residents. This care spectrum spans from primary care to advanced tertiary specialized care. Within this hospital, the department covers an extensive array of services, including pediatric orthopedic trauma and general issues, spinal and foot deformities, and pediatric orthopedic oncology.

Study subjects

The study involved 103 pediatric patients, all aged 12 years and below, representing both genders and various nationalities. These patients underwent a follow-up process involving serial casting at our clinic subsequent to receiving diagnoses between 2015 and 2021. Specifically, the research focused on child patients who received serial casting as part of their treatment plan. Exclusions from the study involved children above the age of 12 and those whose parents opted not to participate.

Data collection

A chart review study was done, and the data were collected by Excel data sheet that includes different variables and risk factors. The variables and risk factors include age at presentation, sex, unilateral/bilateral, types of CTEV to be either typical or atypical (syndromic or associated with other congenital disorders), severity (measured by the Pirani score), number of the applied cast, need for tenotomy procedure (tendoachilles lengthening), Family fistory, the time period needed to relapse, and reason for relapse. The compliance was assessed based on a regular follow-up in the clinic and wearing the foot brace post serial casting.

Statistical analysis

The relationship between the Pirani score and the number of casts for patients was assessed by the Spearman rank correlation coefficient. All the data that were at risk of relapse were analyzed with the use of univariate logistic regression analysis modeling and odds ratios. Unadjusted odds ratios with 95% confidence intervals and p-values were calculated. Reference categorical characteristics were indicated with an odds ratio of 1.00. The value of p < 0.05 was considered significant.

## Results

**Figure 1:**
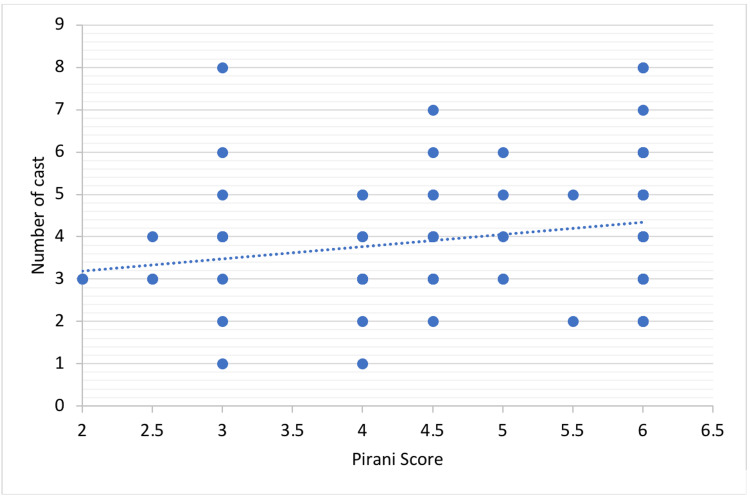
Distribution of Pirani scores in association with the number of casts applied

**Figure 2:**
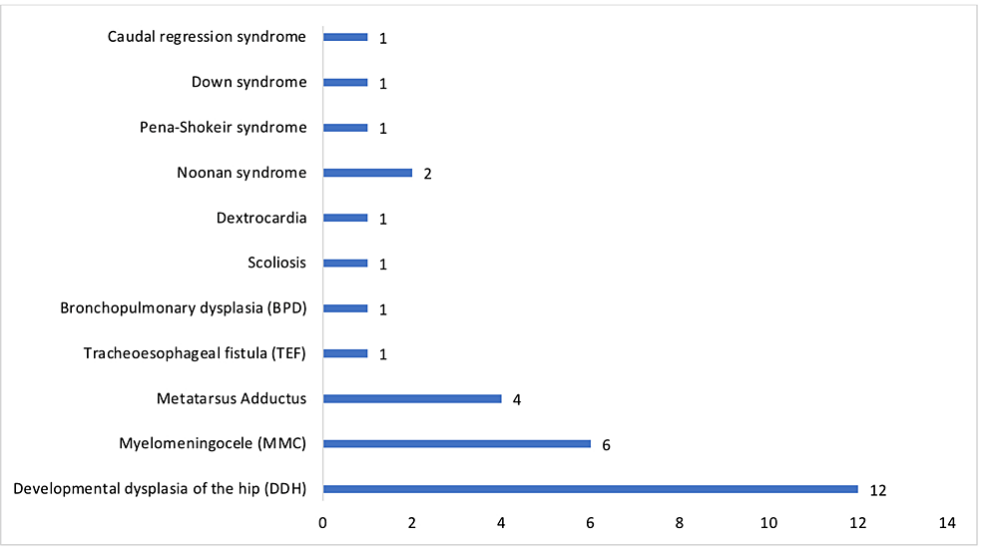
Frequency distribution of the atypical form

The study includes 103 patients with CTEV, and only 22 patients had relapse. The prevalence rate of relapsed cases was 20.4%, and the incidence was 42 per thousand. The average number of casts applied was 4.05 ± 1.37. The average severity of the deformity that was measured by the Pirani score was 4.97 ± 1.21. Table 1 shows the relationship between the Pirani score and the number of casts for the correlation coefficient r = 0.33 with p-value < 0.01, which indicates a weak positive linear correlation. Figure 1 shows the scatter plot of the Pirani scores in association with the number of casts applied. However, the only significant factors in the study were the Pirani score and non-compliance of the brace with a p-value less than 0.05. Table 2 shows the distribution of patients with associated factors related to relapse of CTEV. The relapse was more common in patients above the age of one year at presentation and males more than females. Patients who had bilateral CTEV were more prone to relapse than those with unilateral CTEV. The atypical presentation that was associated with neurological disorders had more relapse compared to those who had atypical presentation, yet without neurological disorders. The Pirani score was one of the main significant risk factors when comparing mild to moderate scores (OR = 0.01; 95%CI = 0.001-0.19). Moreover, patients who underwent tenotomy of tendoachilles had less risk of relapse. Additionally, patients with a positive family history had more prone to relapse than patients with a negative family history. The most common atypical presentation of CTEV was associated with developmental dysplasia of the hip (DDH), followed by MMC. Figure 2 shows the frequency distribution of atypical presentation.

**Table 1:** Spearman rank correlation coefficient

Spearman rank correlation coefficient		Number of casts (4.05 ± 1.37)
Pirani score (4.97 ± 1.21)	r	0.33
	p-value	0.00

**Table 2:** Distribution of patients associated factors related to relapse clubfoot

Factors		Relapse				Total	OR	95% Confidence Interval		P-value
		Yes (n=22)		No (n=81)				Lower Limit	Upper Limit	
		n	%	n	%					
Age (in years)	≤1 (ref.)	20	90.9	71	87.7	91	1.00 1.40	0.28	6.95	0.67
	>1	2	9.1	10	12.3	12				
Gender	F (ref.)	8	36.4	30	37.0	38	1.00 0.97	0.36	2.58	0.95
	M	14	63.6	51	63.0	65				
Side of Clubfoot	Both (ref.)	13	59.1	36	44.4	49	1.00 3.61 1.28	0.73 0.45	17.63 3.68	0.11 0.63
	Lt	2	9.1	20	24.7	22				
	Rt	7	31.8	25	30.9	32				
Type of Clubfoot	Idiopathic (ref.)	19	86.4	79	97.5	98	1.00 0.16	0.02	1.02	0.05
	Syndromic	3	13.6	2	2.5	5				
Typical/Atypical	Atypical (ref.)	6	27.3	22	27.2	28	1.00 1.01	0.34	2.89	0.99
	Typical	16	72.7	59	72.8	75				
Pirani Score	Mild	0	0.0	2	2.5	2	0.01 1.00 0.91	0.00 0.31	0.19 2.62	0.00 0.86
	Moderate (ref.)	6	27.3	23	28.4	29				
	Severe	16	72.7	56	69.1	72				
Number of Casts	≤5 (ref.)	18	81.8	71	87.7	89	1.00 0.63	0.17	2.25	0.48
	>5	4	18.2	10	12.3	14				
Tenotomy	Yes (ref.)	19	86.4	74	91.4	93	1.00 0.59	0.14	2.53	0.48
	No	3	13.6	7	8.6	10				
Family History	Positive (ref.)	3	13.6	4	4.9	7	1.00 3.03	0.62	14.74	0.16
	Negative	19	90.9	77	95.1	96				
Brace Compliance	Yes (ref.)	0	0.0	81	100.0	81	1.00 0.00	0.00	0.01	0.00
	No	22	100.0	0	0.0	22				

## Discussion

CTEV is a congenital foot abnormality. It is one of the most prevalent congenital malformations and manifests in different clinical forms with varying degrees of severity. The consensus supports a number of genetic and environmental risk factors that contribute in varied degrees to the clinical presentations of the condition, even though the exact etiology is still up for debate. CTEV affects 0.5 and two cases per 1,000 births with varying trends of incidence, in particular ethnicities [8]. Males are twice more likely to have CTEV than females [9].

The gold standard treatment of CTEV is the serial casting of the foot, followed by regular corrections with the cast, a method known as the Ponseti technique. Casting can be used up to the age of two; however, it is recommended to start during the first two weeks of life. Depending on how stiff the foot is, five to nine casts are normally needed, and they are replaced every five to seven days [10]. When diagnosed and treated properly, CTEV has good success rates for treatment and overall patient outcomes. Therefore, an optimum long-term outcome is facilitated by early detection and diagnosis in the infant.

**Figure 3:**
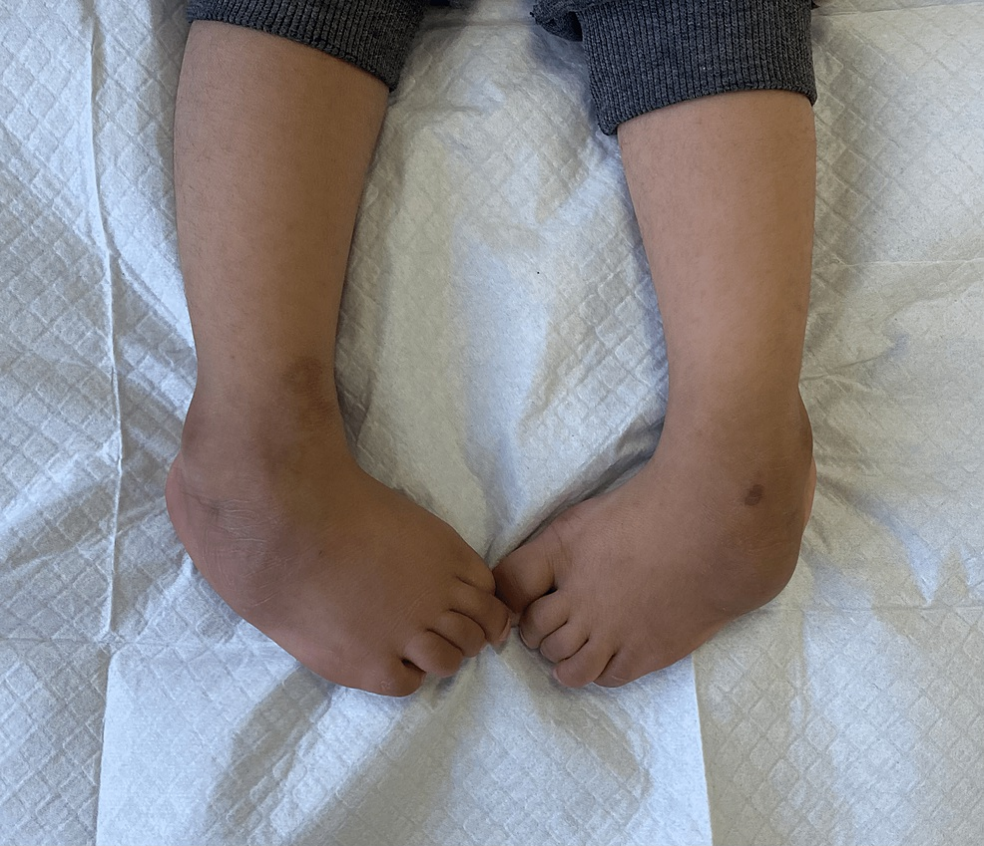
A four-year-old boy who had a relapse after management by the Ponseti method

Non-compliance with the foot brace post serial casting could be attributed to multiple reasons, such as parental educational levels, parental marital status, marital income, number of siblings, the availability of the brace in the hospital especially in the developing country, and atypical presentation of some children [2,11-16]. In contrast, another study did not show any correlation with parental education and income or cultural factors [17]. Figure 3 shows one of the relapsed cases in our clinic at the age of four years.

The estimated time from the last cast applied to the relapse was in the range of four months to two years, with a median age of 18 months. In all 22 patients with a relapsed CTEV, we found that 13 patients had a relapse in less than six months, and nine patients had a relapse in more than six months. In another study, the median age at first relapse was 20 months, and the probability of the relapse increases as the age increases [18]. However, it was recommended in almost all studies to keep bracing until at least the age of four years to avoid relapse [19]. Relapses in this context seem to be associated with the rate of collagen synthesis during foot growth. As a result, premature infants experience rapid relapses, while older infants see a slower recurrence. Relapses are infrequent and less severe in cases of mild club feet with minimal fibrosis, as well as in children with lax ligaments. These relapses arise due to the persistence of factors that caused the deformity. Beyond the age of four, relapses become uncommon, irrespective of whether the deformity was fully or partially corrected [20]. We found that patients who underwent a tenotomy of the Achilles tendon showed a decreased likelihood of relapse, a finding consistent with the report reported by Ponseti [20].

The beginning of the first cast of Ponseti in those who have non-idiopathic atypical neurogenic presentation was delayed, and it took a longer time compared to idiopathic CTEV [14,21]. Eventually, there was not any significance in the correction of the deformity by Ponseti between idiopathic and non-idiopathic CTEV based on the number of casts and the Pirani score [21].

**Figure 4:**
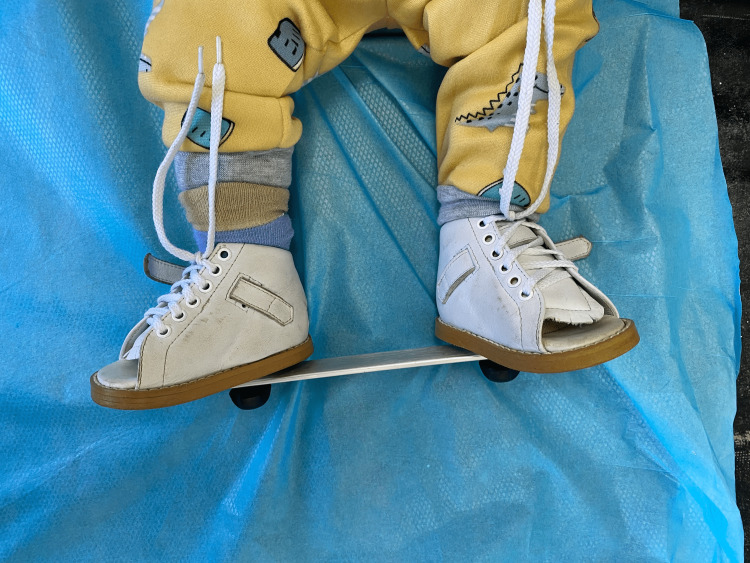
The standard foot abduction brace (Denis brown bar)

Denis brown bar is a type of foot abduction brace that is used commonly after the serial casting of the Ponseti method. However, non-compliance with this kind of brace becomes the main reason for the recurrence in almost all children with CTEV, due to the limitation of movement that is caused by the rigid bar [22]. Recently, a dynamic foot abduction brace has been used and reported in the literature with more advantages over the standard foot brace with less recurrence, less surgical intervention, and fewer skin complications with better compliance and outcomes [22]. Figure 4 shows the standard foot abduction brace that we use in our clinic.

Dimeglio and Pirani scores are two main severity classification scores that are used mostly in determining the severity of CTEV. The accuracy of the severity score in predicting the number of casts and the need for tenotomy has been reported in one study, which shows a slightly better accuracy with the Dimeglio score compared to the Pirani score [23].

Limitations

 There are multiple limitations in the study. The lack of some data in the patient's files, such as the Dimeglio score, parental education and income, and the number of siblings, has led us to address only certain factors. However, the number of patients included in the study could be increased, yet, unfortunately, the documentation system in our hospital has changed multiple times, which led to less comprehensive and accurate file documentation.

## Conclusions

CTEV is a common pediatric disorder in orthopedic practice. The Ponseti method with serial casting has been the gold standard of management, yet there are still a high number of relapse rates reported in the literature. The incidence rate of relapsed cases in our study was reported among nearly one-fifth of cases. The only significant factors in the study were the Pirani score and non-compliance of the brace with a p-value less than 0.05. DDH, followed by MMC, was the most common atypical presentation of CTEV. The correction of deformity by the Ponseti method did not show a significant difference between idiopathic and non-idiopathic CTEV, as assessed by the number of casts and the Pirani score. The dynamic foot brace can be the solution for the high recurrence rate, yet more studies are needed in the future.
